# ICRF193 potentiates the genotoxicity of etoposide

**DOI:** 10.1038/s41598-025-03522-6

**Published:** 2025-06-04

**Authors:** Masato Ooka, Leah Mitchell, Jinghua Zhao, Kouji Hirota, Ruili Huang, Takuya Abe, Menghang Xia

**Affiliations:** 1 Division of Preclinical Innovation, National Center for Advancing Translational Sciences, National Institutes of Health, Bethesda, MD USA; 2 The Graduate School of Science, Tokyo Metropolitan University, Hachioji, Tokyo Japan; 3 Division of Biochemistry, Tohoku Medical and Pharmaceutical University, Sendai, Miyagi Japan

**Keywords:** High throughput screening, Topoisomerase 2, Etoposide, ICRF193: synergistic effect, Genotoxicity, Cytotoxicity, Biochemistry, Cell biology, Chemical biology, Molecular biology

## Abstract

ICRF193 is a catalytic inhibitor of Topoisomerase 2 (TOP2), one of the major targets in cancer therapy. Although ICRF193 has not been approved for clinical use, it has potential implications in chemotherapy. In this study, we aimed to investigate the use of ICRF193 in chemotherapy in co-treatment with other drugs. To identify compounds that have synergistic effects with ICRF193, we optimized a cytotoxicity assay with combinations of ICRF193 in a 1536-well plate format and screened 2678 compounds, including clinically approved and investigational drugs, for their cytotoxicity in the presence and absence of ICRF193. From the screening and confirmation assays, etoposide, a known TOP2-targeting drug, was found to have a synergistic effect with 200 nM ICRF193 across multiple cancer cell lines, including HCT116, MCF7, and T47D. On the other hand, ICRF193 suppressed the toxicity of etoposide at higher concentrations (> 10 µM). In the follow-up studies, we found that ICRF193 and etoposide synergistically induced DNA double-strand breaks and subsequent G2 phase accumulation. Interestingly, this synergistic effect was observed only with etoposide and not with other TOP2 inhibitors in the tested compound library. Taken together, our results indicate that ICRF193 has a specific functional interaction with etoposide that enhances its genotoxic potential.

**Supplementary Information:**

The online version contains supplementary material available at 10.1038/s41598-025-03522-6.

## Introduction

According to a report from the World Health Organization, cancer is the second leading cause of death globally, causing nearly 10 million deaths yearly^1^. Due to its high mortality rate and increasing incidence, various cancer treatments have been developed, though they each come with advantages and disadvantages. Chemotherapy is one of the most common cancer treatments. While many chemotherapy agents, such as cisplatin, induce DNA damage in both normal and cancerous cells, other agents target specific proteins^2,3^.

Topoisomerase 2 (TOP2) is one of the major targets in cancer therapy. TOP2 plays an important role in DNA decatenation in cell growth-dependent processes, such as DNA replication and chromosome segregation. TOP2 decatenates DNA through three sequential steps involving cutting of DNA, passing the DNA strand through the break, and re-ligation of the DNA duplex^4^. TOP2 dysfunction causes the accumulation of catenated DNA, leading to DNA double-strand breaks (DSB) in proliferating cells^5^. Since the inhibition of TOP2 induces DSBs in growing cells, TOP2 has been extensively studied as a target of chemotherapy, resulting in the development of several TOP2 inhibitors and TOP2 poisons^6^. While TOP2 inhibitors prevent the catalytic activity of TOP2, TOP2 poisons stabilize TOP2 on DNA before re-ligation, leading to the formation of a toxic TOP2-DNA cleavage complex (TOP2cc).

ICRF193, a bisdioxopiperazine derivative, is a catalytic inhibitor of TOP2^7^. Although ICRF193 itself does not have a strong antineoplastic effect compared to other TOP2-targeting drugs, its clinical potential has been studied, as it catalytically inhibits TOP2 without a strong genotoxic effect^8,9^. Currently, ICRF193 is mainly used to study the function of TOP2 in laboratory settings. Each TOP2-targeting drug prevents TOP2-mediated DNA decatenation in different ways: etoposide stabilizes TOP2cc before the re-ligation steps, while ICRF193 inhibits the ATPase activity of TOP2, which is responsible for its catalytic activity^10^. ICRF187, another bisdioxopiperazine derivative also known as dexrazoxane, has a structure similar to that of ICRF193. It has been used to treat extravasation of intravenous anthracycline chemotherapy and it is also used for its cardioprotective capability during chemotherapy^11,12^. The majority of TOP2-targeting drugs stabilize TOP2 onto DNA, generating intermediates (i.e., TOP2cc) that are potentially toxic to noncancerous cells. ICRF193 is a valuable option for cancer therapy as an enhancer of other drugs (i.e., lowering the effective concentration of other drugs) due to its catalytic inhibition capability against TOP2. Therefore, we focused on investigating the potential use of ICRF193 in combination with other drugs for cancer therapy.

To explore the potential use of ICRF193 in combination with other drugs, we aimed to identify compounds that have lower IC_50_/EC_50_ values when combined with ICRF193 by screening the National Center for Advancing Translational Sciences (NCATS) Pharmaceutical Collection (NPC), a library of 2678 approved and investigational drugs^13^. Because the NPC library is composed of known drugs, their mechanisms of action have been thoroughly studied. Combining the NPC library compounds and compounds of interest allows us to investigate potential chemotherapy approaches utilizing the synergistic effect of drug combination, where the simultaneous treatment of two compounds results in a higher efficacy than the sum of the separate treatments.

In this study, we first optimized the in vitro cell viability assay with combination treatment of ICRF193 in a 1536-well plate format. We then screened 2678 compounds from the NPC library for their cytotoxicity in the presence and absence of ICRF193. Several compounds showed higher cytotoxicity when combined with ICRF193 and were retested in confirmation studies. From these studies, we found that etoposide had a synergistic effect with ICRF193. Additionally, we uncovered that ICRF193 lowers the EC_50_ value of etoposide for DNA damage induction in several follow up experiments. Interestingly, this synergistic effect was not observed in other TOP2-targeting drugs. These results indicate that ICRF193 enhances the cytotoxic effect of etoposide by increasing its genotoxicity.

## Materials and methods

### Cell culture

HCT116 cells (ATCC, Manassas, VA) were cultured in McCoy’s 5 A medium supplemented with 10% FBS, 100 U/mL penicillin, and 100 µg/mL streptomycin at 37 °C. MCF7 and T47D cells (ATCC) were cultured in Dulbecco’s Modified Eagle Medium containing 10% FBS, 100 U/ml penicillin, and 100 µg/ml streptomycin. TK6 cells (ATCC) were cultured in Roswell Park Memorial Institute 1640 medium supplemented with 10% horse serum, 100 U/ml penicillin, 100 µg/ml streptomycin, and 200 mg/ml sodium pyruvate at 37 °C.

### Cell viability assay

Cell viability assay was conducted using the previously described method with a slight modification^14^. Cells were plated into a white solid bottom 1536-well plate at 250 cells /well in 6 µl. After dispensing, the assay plate was incubated at 37 °C for 5 h for cell attachment. Twenty-three nl of the tested compounds and the positive control (i.e., tetraoctylammonium bromide) were transferred using a Pintool station (Wako Automation, San Diego, CA). The assay plate was incubated at 37 °C for 72 h. Four µl of CellTiter-Glo (Promega, Madison, WI) were added to each well, and the assay plate was incubated at room temperature (RT) for 30 min. The luminescence signal was measured using a ViewLux plate reader (Perkin Elmer, Waltham, MA).

### DNA synthesis assay

DNA synthesis assay was conducted using Click-iT™ EdU Proliferation Assay for Microplates (ThermoFisher, Waltham, MA). HCT116 cells were plated into a black wall/ clear bottom 1536-well plate at 1000 cells/well in 5 µl. The assay plate was incubated at 37 °C overnight. Twenty-three nl of the tested compounds and the positive control (i.e., aphidicolin) were transferred using a Pintool station. The assay plate was incubated at 37 °C for 4 h. One µl of EdU reagent was added to each well and the assay plate was incubated for 1 h at 37 °C. The medium was discarded using a Blue Washer (BlueCatBio, Lebanon, NH). Three µl of the fixation solution was added to each well and the assay plate was incubated for 5 min at RT. The assay plate was washed with wash buffer, followed by adding 2 µl of the 1× Click-iT™ EdU reaction cocktail per well, and the plate was incubated for 30 min at RT. After removal of the Click-iT™ EdU reaction cocktail, three µl of 1.5% BSA blocking solution was added to each well, and the assay plate was incubated for 5 min at RT. The assay plate was washed three times with 4 µl/well of wash buffer, followed by the addition of 3 µl of the Amplex™ UltraRed reaction mixture to each well. The assay plate was incubated for 15 min at RT, protected from light. One µl of 3-times diluted Amplex™ UltraRed stop solution was added to each well to stop the reaction. The fluorescence signal was measured using a PHERASTR plate reader (BMG Labtech, Ortenberg, Germany).

### γH2AX immunostaining assay

Immunostaining was conducted using the previously described method with slight modifications^15^. HCT116 cells were plated at 250 cells per well in 6 µl into a 1536-well clear bottom/black wall plate (Aurora Biosciences, San Diego, CA). The assay plate was incubated for 5 h for cell attachment. Twenty-three nl of the compounds were transferred using a Pintool station. After 24, 48, or 72 h, cells were fixed with 4% paraformaldehyde for 20 min, and the plate was incubated with a permeabilization/blocking/nuclear-staining solution (PBS containing 5% BSA, 0.5% Tween-20, 1 µg/ml Hoechst). Next, cells were treated with the anti-γH2AX mouse monoclonal antibody for 1 h and washed with PBS twice. Then, 2 µl of anti-mouse Alexa488 antibody was added to each well, and the plate was incubated for 1 h at RT. After the incubation, the plate was washed three times with PBS. The images were taken using 490 nm excitation and 550 nm emission for γH2AX and 490 nm excitation and 550 nm emission for Hoechst on the Operetta CLS High-Content Analysis System (Perkin Elmer) with a 10× confocal objective. Images from each well of the assay plate were analyzed using Harmony High-Content Imaging and Analysis Software (Perkin Elmer). The number of foci was counted in each well and the number was normalized by cell number to obtain number of foci per cell.

### Cell cycle analysis

TK6 cells were plated at 2 × 10^5^ cells/1.5 ml/well into a 12-well plate. The cells were treated with ICRF193 and etoposide for 16 h. Cells were washed with ice-cold PBS twice. Then, the cells were fixed with 70% ice-cold ethanol for 20 min and then washed with 0.1% BSA in PBS twice. The cells were resuspended in PBS containing 0.1% BSA, 100 µg/ml RNAse, and 50 µg/ml propidium iodide. The cell cycle profile was analyzed using NovoCyto Quanteon (ACEA Biosciences, Santa Clara, CA).

### Data analysis

Analysis of compound concentration-response data was performed as previously described^16^. Briefly, raw plate reads for each titration point were first normalized relative to the positive control compound (-100%; 92 µM aphidicolin for DNA synthesis, 92 µM tetraoctylammonium bromide for cell viability) and DMSO-only wells (0%) as follows: Activity [%] = (V_compound_ - V_DMSO_)/ (V_DMSO_ - V_pos_) ×100, where V_compound_ indicates the compound well values, V_pos_ indicates the median value of the positive control wells, and V_DMSO_ indicates the median values of the DMSO-only wells. An in-house pattern correction algorithm was then applied to the normalized data using the DMSO-only compound plates at the beginning and end of the compound plate stack. The half maximal inhibitory concentration (IC_50_) for each compound and maximum response (efficacy) values were obtained by fitting the concentration-response curves of each compound to a four-parameter Hill equation. Compounds were categorized into Class 1–4 according to the type of concentration-response curve detected, potency, efficacy, and quality of fit^14^. Class 1–3 curves showed at least one point of activity above the background. Class 4 curves were considered inactive.

## Results

### Assay optimization for co-treatment of ICRF193 in a 1536 well plate format

We aimed to identify compounds that have synergistic effects with ICRF193 in the in vitro primary screening. We hypothesized that if ICRF193 potentiates the compound’s cytotoxicity, the concentration-response curve of the tested compounds will shift to the left (Supplemental Fig. 1A). To screen compounds that have synergistic effects with ICRF193 in a quantitative screening format, which allows us to evaluate tens of thousands of compounds efficiently, we first optimized an in vitro cell viability assay using HCT116, a human colon cancer cell line. To determine the cell number for the screening, we tested several cell densities in the assay wells and calculated the IC_5 − 20_ of ICRF193 and the coefficient of variation (CV) for each condition (Supplemental Fig. 1B). IC_5_ is the concentration of ICRF193 when 5% cell death is observed. CV is calculated as (standard deviation / average) × 100 [%] in DMSO-treated wells. Generally, assays with a CV value lower than 10% are considered to be appropriate. The concentration of IC_5 − 20,_ which was 200 nM in the assay wells, was considered high enough to inhibit TOP2 activity without causing significant cell death (Supplemental Fig. 1B). To confirm that this condition can be used to identify compounds that have synergistic effects with ICRF193, we conducted an in vitro cell viability assay against NU7441, a DNA-PK inhibitor known to be potentiated by ICRF193^17,18^, with dose titrations in the presence or absence of 200 nM ICRF193. The cell viability data showed a higher cytotoxic effect with co-treatment of NU7441 and ICRF193 compared to NU7441-only treatment, suggesting that the compound cytotoxicity potentiation by ICRF193 is detectable in this condition (Supplemental Fig. 1C). Based on the optimization data, the screening condition was set to 250 cells per well with/without 200 nM of ICRF193.

### Identification of four compounds from the primary screening

Following assay optimization, we screened 2678 approved and investigational drugs from the NPC library with concentrations ranging from 0.78 nM to 45.8 µM. The cells were treated with compounds in the presence or absence of 200 nM ICRF193. The efficacy [%] and IC_50_ values were calculated for each tested compound (Supplemental Table 1). To determine active compounds, we used an efficacy cutoff of 40% or more, an IC_50_ cutoff of 5 µM or less, and a concentration-response curve quality cutoff (i.e., r^2^ > 0.9) with ICRF193 co-treatment. The list of compounds was further narrowed down based on two criteria: (1) the compound was cytotoxic in the presence of ICRF193 and was not cytotoxic in the absence of ICRF193, or (2) the compound was cytotoxic in both the presence and absence of ICRF193 with at least a 6-fold IC_50_ difference. Based on these criteria, four compounds, etoposide, trametinib, mebendazole, and oxfendazole, were selected for the confirmation study (Table 1).

**Table 1:** Compounds that increased potency when co-treated with ICRF193 were identified from the primary screening.

Compound	Without ICRF193 IC_50_ (Efficacy)	With ICRF193 IC_50_ (Efficacy)
Trametinib	0.26 µM (92.3%)	0.021 µM (82.8%)
Mebendazole	20.59 µM (55.2%)	0.92 µM (61.9%)
Oxfendazole	14.58 µM (44.2%)	2.06 µM (49.7%)
Etoposide	29.08 µM (66.7%)	2.91 µM (99.1%)

### Confirmation of the synergistic effect of etoposide and ICRF193 for cytotoxicity

**Fig. 1:**
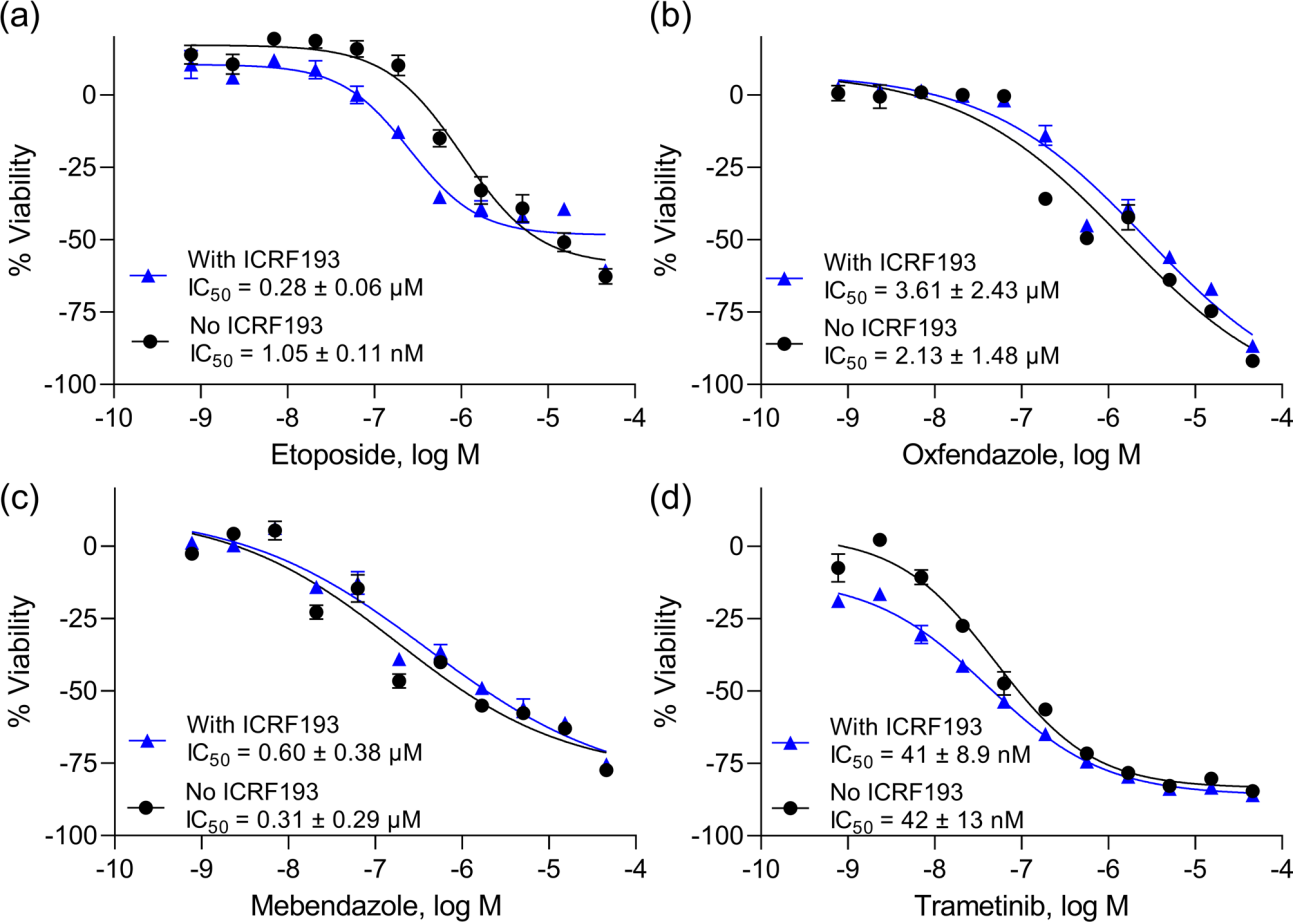
Confirmation study for the selected compounds. The cherry-picked compounds were retested in the confirmation study. The cells were treated with (**a**) etoposide, (**b**) oxfendazole, (**c**) mebendazole, and (**d**) trametinib in the presence or absence of 200 nM ICRF193. The y-axis indicates the viability of the cells after 72 h of treatment. The-x axis indicates the concentration of the indicated compounds. The error bars indicate the standard deviation from three independent experiments.

The compounds were retested for their cytotoxic effects in the presence or absence of ICRF193 in a confirmation study. The IC_50_ values of each compound were compared with and without ICRF193. Among the tested compounds, only etoposide showed a higher potency in the presence of ICRF193 compared to in the absence of ICRF193. IC_50_ values were 278 nM in the presence of ICRF193 and 1.05 µM in the absence of ICRF193, confirming the primary screening results (Fig. 1a). This result is consistent with a previous report showing that ICRF193 enhanced the cytotoxic effect of etoposide at low concentrations^19^. The other tested compounds did not show potentiated cytotoxicity in the presence of ICRF193 in the confirmation study (Fig. 1b–d). Interestingly, the other 13 TOP2-targeting drugs in the NPC library, such as teniposide, did not show an enhanced effect with ICRF193 (Supplemental Table 1). Although three TOP2 inhibitors, aclarubicin, idarubicin, and daunorubicin, showed slightly higher potency when combined with ICRF193, the difference was below the cutoff criteria. To confirm that they are not enhanced by ICRF193, we retested these three compounds in the cell viability assay. As suggested in the primary screening, we did not see potentiation of these three compounds by ICRF193 (Table 2).

**Table 2:** IC_50_ and efficacy of TOP2-targeting drugs with or without ICRF193.

	Without ICRF193		With ICRF193	
	IC_50_ [nM]	Efficacy [% inhibition]	IC_50_ [nM]	Efficacy [% inhibition]
Aclarubicin	12.36 ± 2.23	86.82 ± 0.67	15.49 ± 1.98	83.01 ± 0.94
Idarubicin	105.83 ± 59.96	83.75 ± 0.48	199.42 ± 58.73	84.81 ± 0.29
Daunorubicin	31.49 ± 8.9	90.34 ± 1.3	47.07 ± 5.41	89.74 ± 1.13

**Fig. 2:**
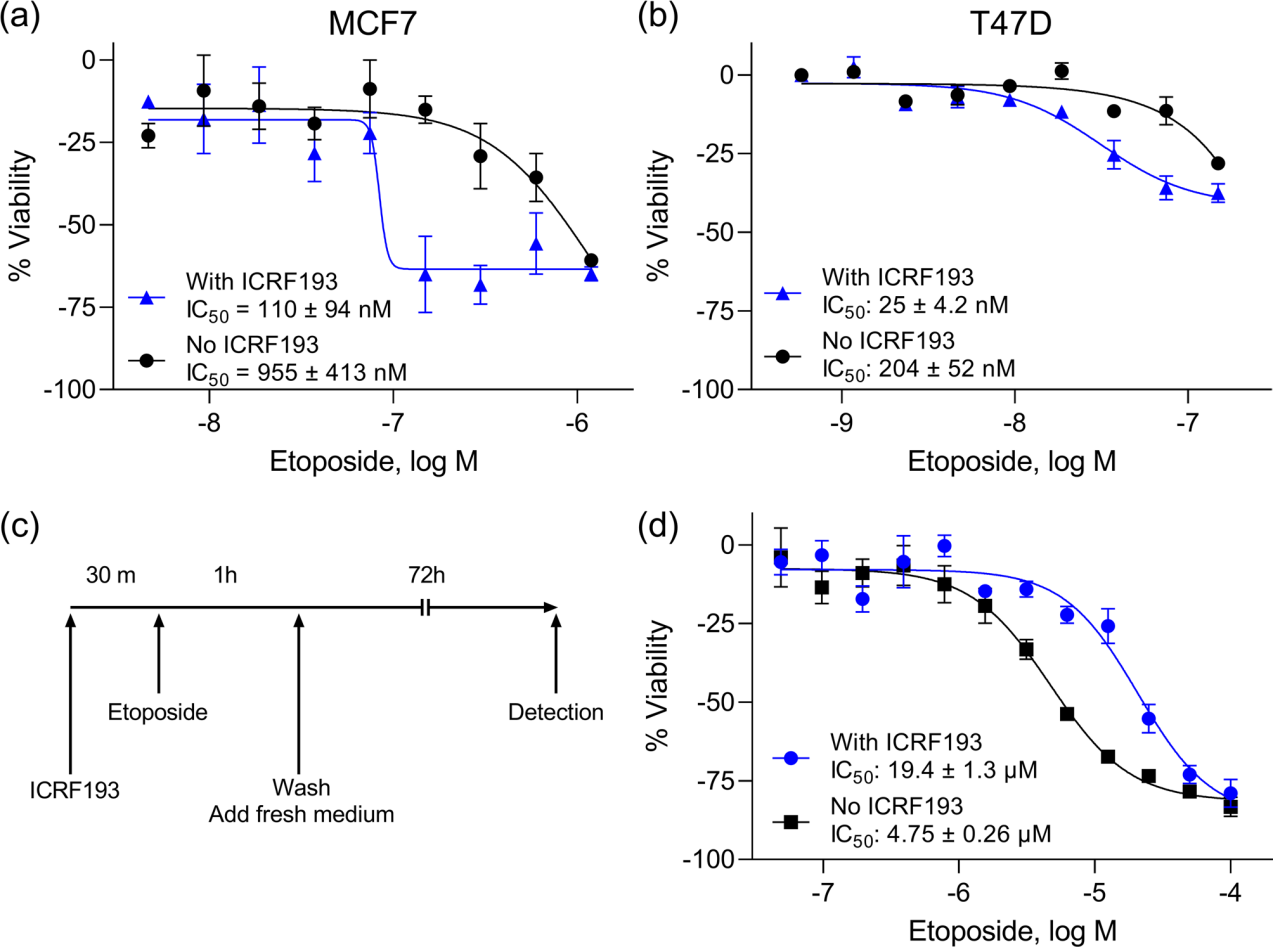
Potentiation effects in other cancer cells and pre-treatment of ICRF193. Human breast cancer cells, (**a**) MCF7 and (**b**) T47D cells, were treated with etoposide in the presence or absence of 200 nM ICRF193 for 72 h. (**c**) The timeline of the pre-treatment experiment. First, the cells were treated with 10 µM ICRF193 for 30 min, then several concentrations of etoposide were added to each well, followed by 1 h incubation. After washing, the cells were cultured for 72 h in fresh medium. (**d**) Cell viability assay result for etoposide with or without pre-treatment with 10 µM ICRF193 treatment. The-y axis indicates the percent viability of the cells. The x axis indicates the concentrations of etoposide. The error bars indicate the standard deviation from three independent experiments.

To evaluate this potentiation in other cell types, we tested the co-treatment of ICRF193 and etoposide in two human breast cancer cell lines, MCF7 and T47D. The enhanced effect was also observed in these cell lines. In MCF7 cells, the IC_50_ values were 110 nM with ICRF193 and 955 nM without ICRF193 (Fig. 2a). In T47D cells, the IC_50_ values were 25 nM with ICRF193 and 204 nM without ICRF193 (Fig. 2b). These results indicate that the enhanced cytotoxic effect is not specific to HCT116 cells and occurs within other cell types. It is known that complete inhibition of the catalytic activity of TOP2 reduces the cytotoxicity of etoposide by preventing the generation of TOP2cc^20,21^. To confirm this effect, we pre-treated HCT116 cells with 10 µM ICRF193 for 30 min and then etoposide for 1 h (Fig. 2c). We found that high concentrations (i.e., 10 µM) of ICRF193 suppressed etoposide toxicity (Fig. 2d), which was consistent with a previous report^21^. Our results indicate that ICRF193 enhances the cytotoxicity of etoposide at low concentrations, but it attenuates the cytotoxicity of etoposide at high concentrations. This synergistic effect was also observed in the co-treatment of etoposide and ICRF187, an analog of ICRF193, at 10, 20, 40 µM (Supplemental Fig. 2).

### Evaluating the genotoxicity of etoposide when co-treated with ICRF193

Although the synergistic effect of ICRF193 and etoposide on cytotoxicity has been reported, the underlying mechanism has not been studied. To investigate the mechanism of cytotoxicity potentiation, we evaluated the genotoxic potential of etoposide in combination with ICRF193. HCT116 cells were treated with etoposide and ICRF193 for 4 h. Their effect on DNA synthesis was measured using a 5-ethynyl-2´-deoxyuridine (EdU) incorporation assay. EdU, a nucleoside analog, is incorporated into genomic DNA during DNA replication. Subsequent measurement of EdU incorporation allows for an estimation of DNA replication activity within cells. The IC_50_ values for the inhibition of DNA synthesis by etoposide decreased by more than 2-fold when co-treated with 240 nM or 480 nM of ICRF193 compared to the IC_50_ value without ICRF193 (Table 3). This potentiation was not observed with 980 nM ICRF193 (Table 3). This result is consistent with the cell viability assay, where ICRF193 potentiated etoposide at 200 nM but inhibited it at 10 µM.

**Table 3:** IC_50_ values of etoposide co-treated with/without ICRF193 in DNA synthesis assay.

ICRF193 concentrations [nM]	IC_50_ values of etoposide [µM]	Fold change
955	15.5 ± 8.4	1.09
477	7.9 ± 1.1*	2.14
239	6.7 ± 0.5*	2.53
119	9.14 ± 4.0	1.84
59.7	8.6 ± 1.6	1.96
29.8	12.3 ± 4.6	1.37
0	16.9 ± 5.4	

The fold change was calculated based on the IC_50_ value of etoposide without ICRF193. *p-value < 0.05.

To further analyze the genotoxic effect of etoposide in the presence and absence of ICRF193, we evaluated the amount of DNA damage by measuring the number of γH2AX foci per cell. γH2AX is a phosphorylated form of histone H2AX, representing DNA damage^22^. HCT116 cells were treated with etoposide and ICRF193 for 24, 48, or 72 h. The number of γH2AX foci per cell was measured after co-treatment with ICRF193 and etoposide. Potentiation of etoposide genotoxicity was observed after 24 h, 48 h, and 72 h of treatment at lower concentrations, while ICRF193 inhibited the etoposide-induced genotoxicity at higher concentrations (e.g., 15.3 µM). Similar to the cell viability assay and DNA synthesis assay, the potentiation was only observed with low concentrations of ICRF193 (i.e., < 1 µM) and the genotoxicity of etoposide was suppressed with higher concentrations of ICRF193 (Supplemental Table 2). We conducted cell cycle analysis using TK6 cells to see the effect of ICRF193 and etoposide on cell cycle distribution. Etoposide increased accumulation in the G2 phase at 10 nM and 20 nM (Fig. 4a–c). ICRF193 itself did not have a detectable effect at 10 nM (Fig. 4a and d). When cells were treated with 10 nM ICRF193 together with either 10 nM or 20 nM etoposide, the population of cells in the G2 phase further increased (Fig. 4e and f). The cell cycle arrest in the G2 phase suggests the activation of the G2/M checkpoint, which is an indicator of unrepaired DNA damage. This result is consistent with the aforementioned results. Taken together, our results indicate that ICRF193 enhances the cytotoxicity of etoposide by increasing etoposide-induced DNA damage in cells.

## Discussion

**Fig. 3:**
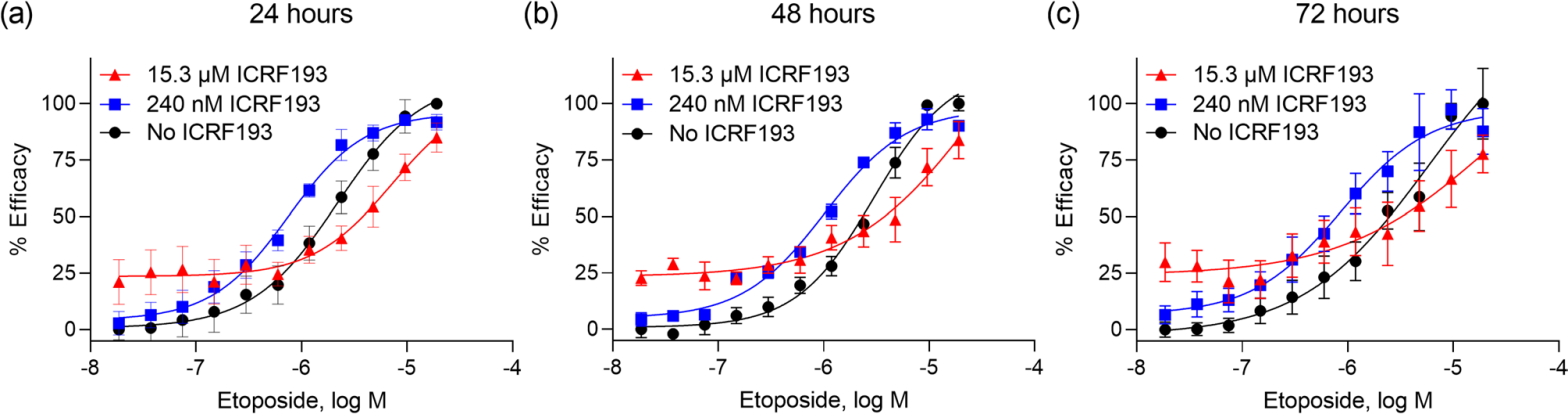
Evaluation of DNA damage induced by combination treatment of etoposide and ICRF193. The cells were treated with etoposide in the presence or absence of ICRF193. The number of foci was counted in each condition. Representative concentration-response curves of etoposide with the indicated concentration of ICRF193 after (**a**) 24 h, (**b**) 48 h, or (**c**) 72 h of treatment. The-y axis indicates the mean number of foci per cell. The-x axis indicates the concentration of etoposide.

**Fig. 4:**
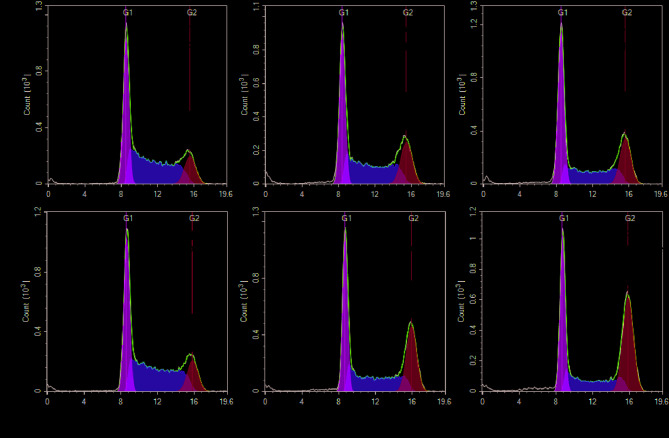
Cell cycle profiling after individual or co-treatment of etoposide and ICRF193. TK6 cells were treated with etoposide and/or 10 nM ICRF193. (**a**) No ICRF193 / No etoposide, (**b**) No ICRF193 / 10 nM etoposide, (**c**) No ICRF193 / 20 nM etoposide, (**d**) 10 nM ICRF193 / No etoposide, (**e**) 10 nM ICRF193 / 10 nM etoposide, and (**f**) 10 nM ICRF193 / 20 nM etoposide. DNA was stained with propidium iodide (PI). The x-axis indicates the intensity of PI, reflecting the amount of DNA. The y-axis indicates the number of cells in the histogram.

In this study, we optimized a cell viability assay in a 1536-well plate format to identify compounds that have synergistic effects with ICRF193 from a collection of 2678 compounds. We discovered that etoposide had higher cytotoxicity when combined with 200 nM ICRF193. This potentiation was further studied in several follow-up assays. We observed that ICRF193 potentiated etoposide cytotoxicity in two breast cancer cell lines, MCF7 and T47D. The potency of etoposide was increased by approximately 9-fold in these two cell lines when co-treated with 200 nM ICRF193 (Fig. 2). Although the fold-change was not as high as in the cell viability assay, this enhanced inhibitory effect was also observed in the DNA synthesis assay (Table 3). The γH2AX immunostaining assay suggested that etoposide induced DNA damage at lower concentrations in the presence of ICRF193 (Fig. 3). This result was further supported by the cell cycle analysis, which showed a more pronounced accumulation of cells in the G2 phase when co-treated with ICRF193 and etoposide (Fig. 4).

The NPC library includes more than 2000 approved and investigational drugs, which can be used for drug repurposing (i.e., drug repositioning). In this study, we screened the NPC library to identify compounds that have synergistic effects with ICRF193. The assay was optimized in 1536-well plate format and validated using NU7441 (Supplemental Fig. 1C). ICRF193 was developed as a TOP2 inhibitor for potential use in cancer therapy^23^. Due to its low genotoxicity compared to other cancer drugs, ICRF193 is commonly used for TOP2-related research studies instead of clinical cancer treatment^24^. We aimed to find a potential clinical use for ICRF193 by combining it with other drugs. From the primary screening, we identified four compounds with potential synergistic effects with ICRF193, including etoposide, trametinib, mebendazole, and oxfendazole. Among these compounds, only etoposide was confirmed for its enhanced potency with ICRF193 in the confirmation study. Although we could not identify a novel compound that has a synergistic effect with ICRF193 in the NPC library, this method can be applied to another compound library to identify such compounds to explore the potential clinical use of ICRF193.

Even though there are several DNA-damaging reagents, including other TOP2-targeting drugs, in the NPC library, only etoposide showed a significant synergistic effect with ICRF193. Based on the chemical structure, TOP2-targeting reagents are classified into several categories: bisdioxopiperazines, anthracyclines, anthracenediones, and epipodophyllotoxins^25,26^. The NPC library contains 2 bisdioxopiperazines, 7 anthracyclines, 2 anthracenediones, and 2 epipodophyllotoxins. Etoposide is classified as an epipodophyllotoxin. Among these 13 compounds, only etoposide showed an enhanced effect with ICRF193. Interestingly, even the other epipodophyllotoxin, teniposide, did not show an enhanced effect in our study despite its similar structure to etoposide (Supplemental Table 1). Although daunorubicin showed an enhanced effect with an 8.9-fold change in IC_50_ value from the primary screening, it was not selected for the confirmation study due to its poor curve fit (r^2^ < 0.9). Aclarubicin and idarubicin hydrochloride also showed an enhanced effect with IC_50_ fold-changes of 2.5 and 2.8 in the primary screening, respectively. To clarify their relationship with ICRF193, we conducted a confirmation study for these three compounds, but none of them had a synergistic effect with ICRF193 (Table 2). These results indicate that there might be a specific functional interaction between ICRF193 and etoposide. Several studies reported that ICRF193 inhibits the ATPase activity of TOP2 by binding the ATPase domain. TOP2 poisons stabilize the TOP2-DNA complex after the ATP-dependent catalytic activity. Therefore, ICRF193 suppresses the effect of TOP2 poisons^27,28^. On the other hand, it is also reported that ICRF193 enhances the cytotoxic effect of etoposide at lower concentrations^19^. Our results are consistent with these reports, indicating that ICRF193 has two modes of action: inhibiting etoposide at high concentrations and enhancing etoposide-induced toxicity at low concentrations. In the clinical setting, the concentrations of ICRF187, an analog of ICRF193, and etoposide are approximately 300 µM and 4 µM at the peak plasma concentrations, respectively^29,30^. In the cell viability assay with co-treatment of ICRF187 and etoposide, we observed a synergistic effect between them. ICRF187 showed a synergistic effect with etoposide at 10–40 µM (Supplemental Fig. 2), which was much higher than that of ICRF193 (200 nM). Although they may act synergistically after infusion, further studies are needed to evaluate their effects in vivo.

Despite the first report in 1996, there are no available studies reporting the detailed mechanism behind the synergistic cytotoxic effect of ICRF193 and etoposide. Our γH2AX immunostaining assay indicated that etoposide induces DNA damage at lower concentrations when combined with ICRF193 at < 1 µM, while the DNA damage induction by etoposide was suppressed by ICRF193 at > 1 µM (Supplemental Table 2). We observed that the cytotoxicity of etoposide decreased when cells were pre-treated with a higher concentration of ICRF193 (Fig. 2d). Since ICRF193 inhibits the requirement of TOP2, this result indicates that the recruitment of TOP2 on DNA is essential for the synergistic effect. ICRF193 binds to the TOP2 ATPase domain, containing the ATP-binding site, directly involved in clamp closure, where TOP2 binds to DNA^10^. One possible explanation for the potentiation is that a low concentration of ICRF193 partially prevents TOP2 catalytic activity and increases the frequency of catenated DNA, leading to more TOP2 recruitment to the loci. Since low concentrations of ICRF193 may not completely inhibit TOP2, TOP2 can still be recruited and stabilized by etoposide. Another possible explanation is that ICRF193 binds to TOP2 after etoposide stabilizes TOP2 on DNA, resulting in a more stable TOP2cc by keeping the clamp closed. Further studies are needed to clarify the detailed mechanisms underlying the synergistic effects between ICRF193 and etoposide.

In this study, we optimized and validated a quantitative high-throughput cell viability assay in combinations with ICRF193 treatment and screened over 2000 clinically approved and investigational drugs to identify compounds that show a higher efficacy when co-treated with ICRF193. Through the primary screening and confirmation study using colon cancer cells (i.e., HCT116), we found that etoposide became more cytotoxic when co-treated with ICRF193. Interestingly, other TOP2-targeting drugs did not show a synergistic effect with ICRF193, indicating the specific functional interaction between ICRF193 and etoposide. This enhanced effect was also observed in breast cancer cells (i.e., MCF7 and T47D). We further investigated the cellular events in which ICRF193 enhances etoposide. We found that ICRF193 enhanced the cytotoxicity of etoposide by increasing etoposide-induced DNA damage in cells through γH2AX immunostaining, DNA synthesis, and cell cycle profiling assays. Taken together, our study identified the synergistic cytotoxic effect of etoposide and ICRF193. We revealed that the combination treatment of ICRF193 and etoposide showed higher cytotoxicity by synergistically inducing DNA damage. In addition, we found that this synergistic relationship is only observed between etoposide and ICRF193, but not in the other tested drugs, including several TOP2-targeting drugs.

## Electronic supplementary material

Below is the link to the electronic supplementary material.

Supplementary Material 1

Supplementary Material 2

## Data Availability

All data generated or analyzed during this study are included in this published article.
