# Supplementary material for: ICRF193 potentiates the genotoxicity of etoposide
Source: Sci Rep. 2025 Jun 4;15:19518. doi: 10.1038/s41598-025-03522-6 (PMC12137928; doi:10.1038/s41598-025-03522-6)
Supplement: Supplementary file 2 — Supplementary Material 2 [file 41598_2025_3522_MOESM2_ESM.pdf]

Supplemental Fig. 1. Assay strategy and optimization

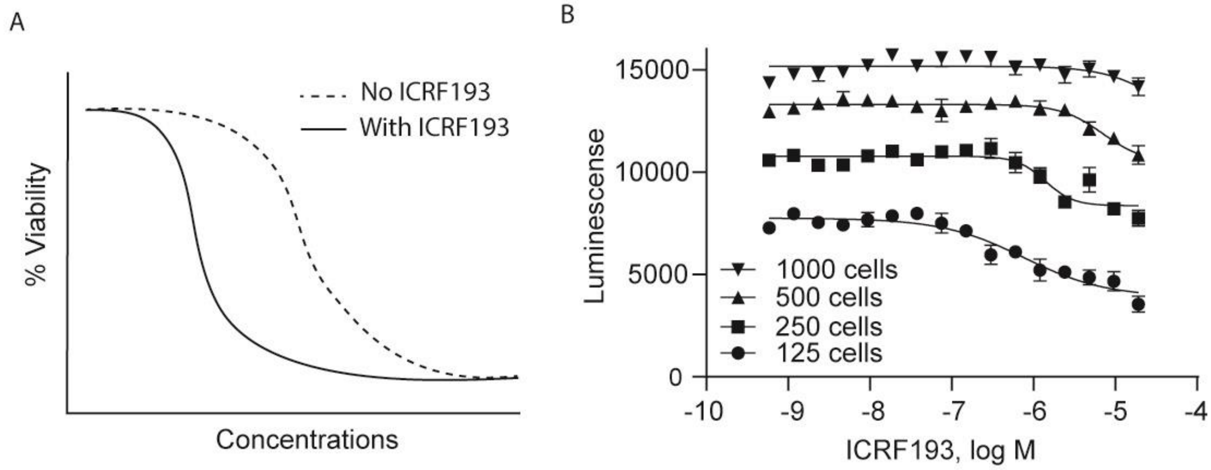

	CV	IC$_5$ [M]	IC$_{10}$ [M]	IC$_{20}$ [M]
125 cells/well	11.3%	2.5×10^{-8}	6.0×10^{-8}	1.5×10^{-7}
250 cells/well	4.4%	2.5×10^{-8}	4.1×10^{-7}	6.9×10^{-7}
500 cells/well	5.6%	1.3×10^{-6}	1.9×10^{-6}	3.1×10^{-6}
1000 cells/well	4.4%	3.4×10^{-6}	6.1×10^{-6}	1.1×10^{-5}

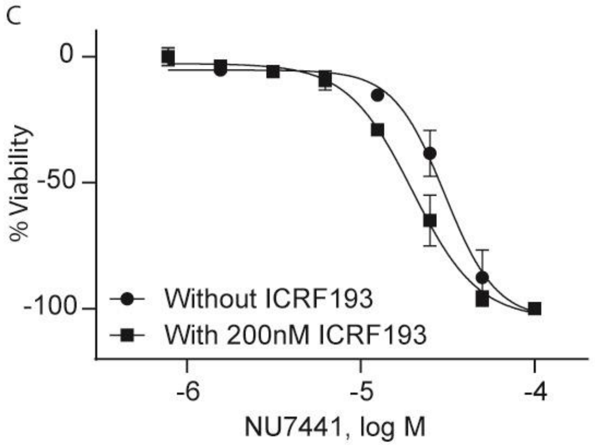

(A)The ideal curve pattern of the compound that have synergistic effect with ICRF193. The curve shifts to the left when cotreated with ICRF193. (B) Cell number optimization. The several density of HCT116 cells were plated and treated with ICRF193. Coefficient variation and IC5-20 was calculated for each condition. (C) Cell viability assay for NU7441 in the presence and absence of 200nM ICRF193.

Supplemental Fig. 2. Cell viability result with cotreatment of ICRF187 and etoposide

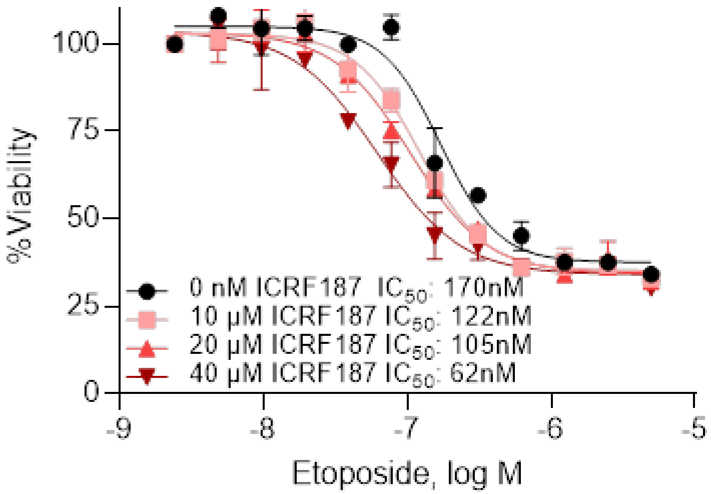

Cells were treated with various concentrations of ICRF187 and etoposide for 72 hours. The x-axis indicates the concentration of etoposide and the y-axis indicates the percentage viability.
